# Gum Elastic in the Treatment of Irregularity of the Teeth

**Published:** 1850-10

**Authors:** 


*Gum Elastic in the Treatment of Irregularity of the Teeth.*—The substitution of gum elastic for silk, and, indeed, every other description of ligature in the treatment of irregularity of the teeth, has been attended with the most satisfactory and happy results. Its great superiority consists in the steady and constant traction which it exerts upon the deviating tooth. The usual method of employing it, consists in tying a ligature of silk to each end of a piece of gum elastic of the requisite size and length, leaving both ends of each sufficiently long to be secured to the fixed point and the tooth, the position of which is to be changed. But it will be seen from an extract of a letter, which we publish in another part of the Journal, from Dr. E. G. Tucker, of Boston, that he has had it prepared expressly for the purpose, superceding altogether the necessity of ligatures of silk. It consists of hollow tubes, varying in size and in the thickness of their walls, but for a description of the manner of using them, we refer the reader to what Dr. Tucker says upon the subject. We have used them, and can recommend them with confidence to the profession.

